# Supplementary material for: Evolutionary dynamics of FoxQ2 transcription factors across metazoans reveals three ancient paralogs
Source: Commun Biol. 2025 Dec 21;9:98. doi: 10.1038/s42003-025-09368-y (PMC12828049; doi:10.1038/s42003-025-09368-y)
Supplement: Supplementary file 1 — Supplementary Information [file 42003_2025_9368_MOESM1_ESM.pdf]

Evolutionary dynamics of FoxQ2 transcription factors across metazoans reveals three ancient paralogs

Giacomo Gattoni*, Che-Yi Lin, Joshua R York, Colin Shew, Daniel Keitley, Carole LaBonne, Jr-Kai Yu, J Andrew Gillis, Elia Benito-Gutiérrez*

* corresponding authors: gg2900@columbia.edu; eb647@cam.ac.uk

Supplementary Figures 1-11

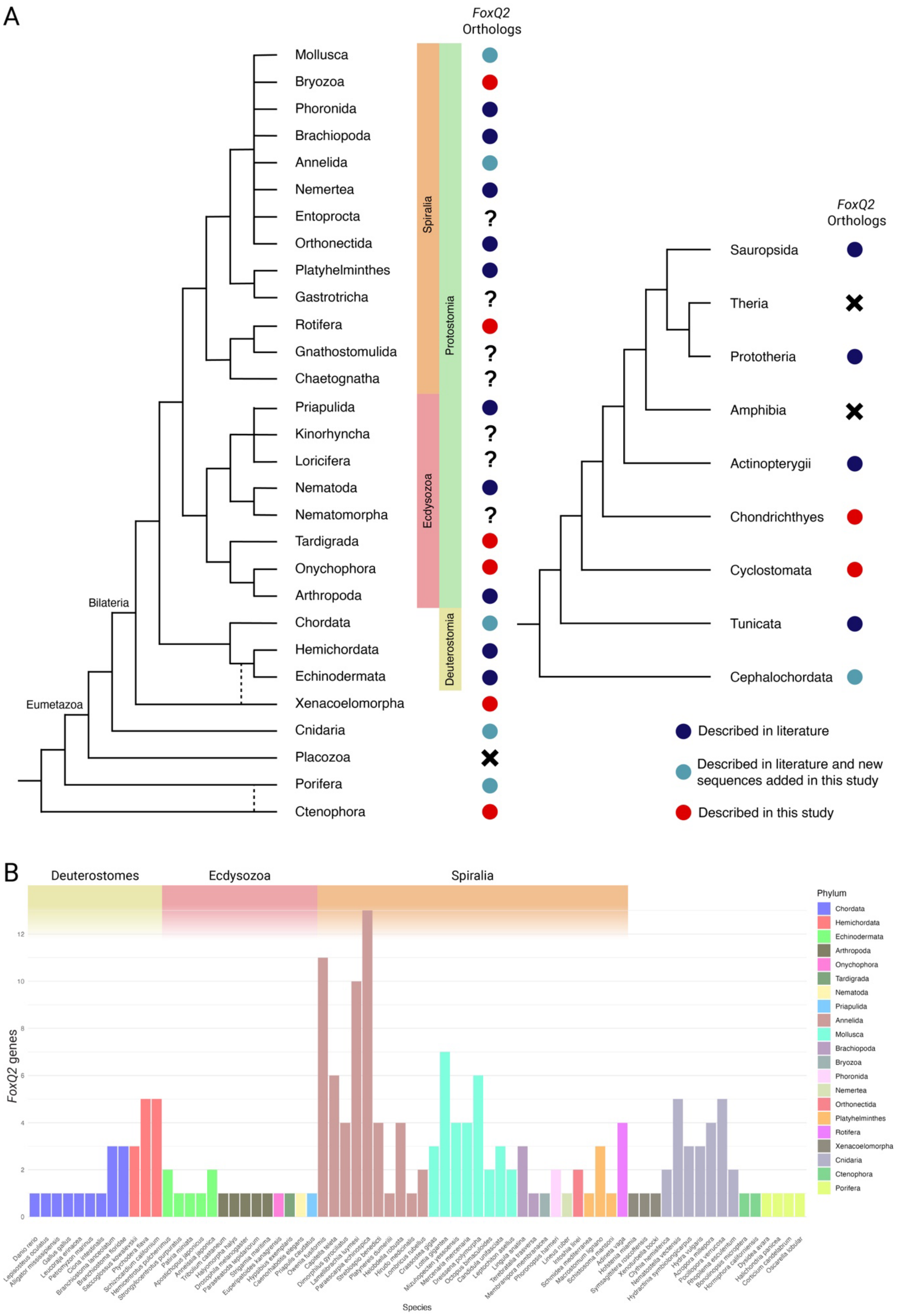
A
FoxQ2 Orthologs
Mollusca
Bryozoa
Phoronida
Brachiopoda
Annelida
Nemertea
Entoprocta
Orthonectida
Platyhelminthes
Gastrotricha
Rotifera
Gnathostomulida
Chaetognatha
Priapulida
Kinorhyncha
Loricifera
Nematoda
Nematomorpha
Tardigrada
Onychophora
Arthropoda
Chordata
Hemichordata
Echinodermata
Xenacoelomorpha
Cnidaria
Placozoa
Porifera
Ctenophora
Spiralia
Ecdysozoa
Protostomia
Deuterostomia
Bilateria
Eumetazoa
FoxQ2 Orthologs
Sauropsida
Theria
Prototheria
Amphibia
Actinopterygii
Chondrichthyes
Cyclostomata
Tunicata
Cephalochordata
Described in literature
Described in literature and new sequences added in this study
Described in this study
B
Deuterostomes
Ecdysozoa
Spiralia
FoxQ2 genes
Species
Phylum
Chordata
Hemichordata
Echinodermata
Arthropoda
Onychophora
Tardigrada
Nematoda
Priapulida
Annelida
Mollusca
Brachiopoda
Bryozoa
Phoronida
Nemertea
Orthonectida
Platyhelminthes
Rotifera
Xenacoelomorpha
Cnidaria
Ctenophora
Porifera

Supplementary Fig. 1. Conservation of *FoxQ2* genes but variation in copy number across metazoans.

A. Schematic tree showing the phylogenetic relationship between animal phyla, with a focus on chordate evolution. *FoxQ2* orthologs can be identified in most phyla investigated (circles) but appear to be lost in specific lineages (crosses). The color of the circles indicates whether *FoxQ2* sequences for specific phyla were described in the literature (blue), discovered in this work (red), or present in the literature but found for new species in this study (teal). **B.** Barplot indicating the variation in copy number of *FoxQ2* genes (y axis) in each of the 70 species considered in this study (x axis). Bars are colored by phylum.

```
Dre_FoxQ2      KPAQSYIALISMAILDSDEKKLLL DIYQWIMDHYPYFKSK----DKNWRNSVRHNLSLNECFIKAGRSDNGKGHFWAIHPANFQDFSNGDYH-RRRAR
Ler_FoxQ2      KPAMSYIALIAKAILTSPSKRLNLGSIYKYIEETFPYYKGR----GQGWRNSVRHNLSLNECFIKAGRCEDGKGNYWSIHPSNLDDFSKGDFRQRRRCR
Pma_FoxQ2      KPRLSYTALIANAILSSRDRRLNLSSIYSWIEERYPFYGRQDRRAARGWRNSVRHNLSLNECFVKVGRCEDGKGNYWGIHEHHVEAFGRGDFR-LNLGS
Cin_FoxQ2      KPERPYVGLIAEAILDSEAKRLSLGQIYQYLEAKYLYFKLR----RGGWKNSIRHNLSLNHCFIKVGRCEDGKGNYWTIHPSYEPAFQRGDFKWRRLSR
Bla_FoxQ2a     KPRHSYIALIAMAIMSSKDKRLLLGDIYQWIMDNFPFYRNN----ERSWRNSIRHNLSLNDCFIKAGRSQDGKGNYWAIHPANMDDFSRGDFH-RRRAR
Bla_FoxQ2b     KPSHSYIGLIAMAIMSSKDKKLVLSDIYKYILDNYPYFRNR----GPGWRNSIRHNLSLNDCFVKMGRSANGKGHYWAVHPANADDFAQGDFR-RRRAQ
Bla_FoxQ2c     KPPLSYIALIAKAILGSPAKRLSLGSIYQYITDNYPYYQNR----GQGWRNSVRHNLSLNDCFIKAGRCEDGKGNYWAIHPANIEDFARGDFRQRRRSR
Aja_FoxQ2-1    KPIFSYIALIAKAILNSSESRLLLSDIYQYIMDNYPYYRNN----DRSWRNSIRHNLSLNECFIKSGRSNDGRGHFWAIHPANVEDFMRGDYR-RRRAR
Aja_FoxQ2-2    KPNHSYIALIAMAIMNSPDKKLILSGIYQYILDNYPYFRTR----GPGWRNSIRHNLSLNDCFVKICRSANGKGHFWAIHPANFHDFSKGDFR-RRRAQ
Dme_FoxQ2      KPQHSYIGLIAMAILSSTDMKLVLSDIYQYILDNYPYFRSR----GPGWRNSIRHNLSLNDCFIKSGRSANGKGHYWAIHPANMEDFRKGDFR-RRKAQ
Cgi_FoxQ2-1    KPPHSYIALISMAILSTSDRKMLLSDIYQYVMDNFPFYNNK----EKAWRNSIRHNLSLNECFVKNGRADNGKGNFWSIHPA VEDFARGDFR-RRQAR
Cgi_FoxQ2-2    KPALSYIALIAKSILESSQKRLSLGSIYSWIEKNYPYYQNR----GQGWRNSVRHNLSLNDCFIKAGRCEDGKGNYWAIHPANIQDFMRGDFRQRRRSR
Cgi_FoxQ2-3    KPNHSYIGLIAMAILSSRDKKLVLSDIYQWILDNYPYFRTR----GPGWRNSIRHNLSLNDCFIKSGRSANGKGHYWAIHPANIDDFQKGDFR-RRRAQ
Hmi_FoxQ2      KPNHSYIGLISMAILSSPEKKLVLSEIYQYILENYAYFRTK----GPGWRNSIRHNLSLNDCFVKAGRSANGKGHYWAIHSA IDDFSKGDFR-RRHAQ
```

Supplementary Fig. 2. Broad conservation of predicted secondary structure of the Forkhead domain in representative bilaterian species.

Amino acids predicted to form alpha-helices are highlighted in yellow, those forming beta-sheets are highlighted in cyan.

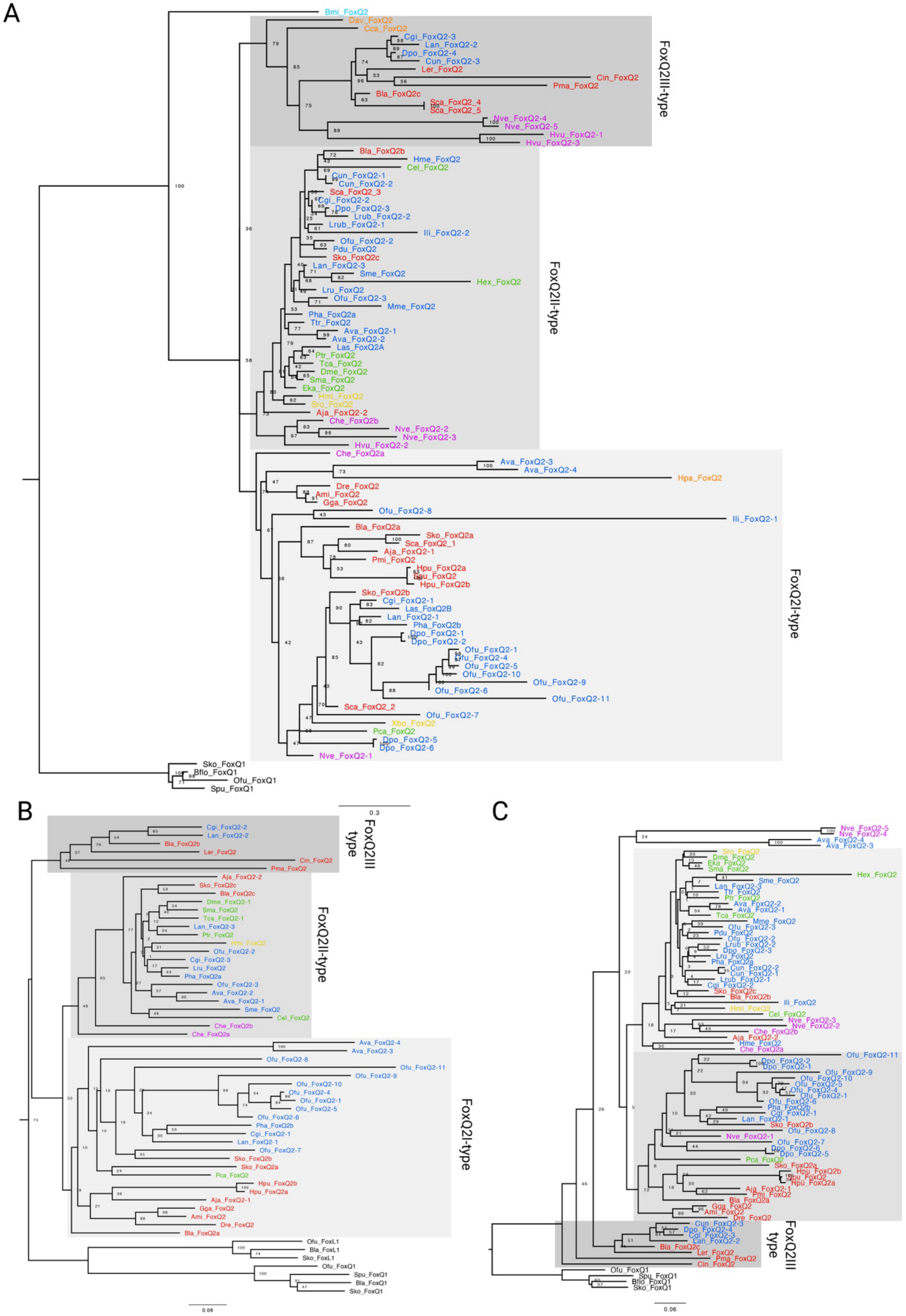

A
FoxQ2III-type
FoxQ2II-type
FoxQ2I-type
Bmi_FoxQ2
Dav_FoxQ2
Cca_FoxQ2
Cgi_FoxQ2-3
Lan_FoxQ2-2
Dpo_FoxQ2-4
Cun_FoxQ2-3
Ler_FoxQ2
Cin_FoxQ2
Pma_FoxQ2
Bla_FoxQ2c
Sca_FoxQ2_4
Sca_FoxQ2_5
Nve_FoxQ2-4
Nve_FoxQ2-5
Hvu_FoxQ2-1
Hvu_FoxQ2-3
Bla_FoxQ2b
Hme_FoxQ2
Cel_FoxQ2
Cun_FoxQ2-1
Cun_FoxQ2-2
Sca_FoxQ2_3
Cgi_FoxQ2-2
Dpo_FoxQ2-3
Lrub_FoxQ2-2
Lrub_FoxQ2-1
Ili_FoxQ2-2
Ofu_FoxQ2-2
Pdu_FoxQ2
Sko_FoxQ2c
Lan_FoxQ2-3
Sme_FoxQ2
Hex_FoxQ2
Lru_FoxQ2
Ofu_FoxQ2-3
Mme_FoxQ2
Pha_FoxQ2a
Ttr_FoxQ2
Ava_FoxQ2-1
Ava_FoxQ2-2
Las_FoxQ2A
Ptr_FoxQ2
Tca_FoxQ2
Dme_FoxQ2
Sma_FoxQ2
Eka_FoxQ2
Hmi_FoxQ2
Sro_FoxQ2
Aja_FoxQ2-2
Che_FoxQ2b
Nve_FoxQ2-2
Nve_FoxQ2-3
Hvu_FoxQ2-2
Che_FoxQ2a
Ava_FoxQ2-3
Ava_FoxQ2-4
Hpa_FoxQ2
Dre_FoxQ2
Ami_FoxQ2
Gga_FoxQ2
Ofu_FoxQ2-8
Ili_FoxQ2-1
Bla_FoxQ2a
Sko_FoxQ2a
Sca_FoxQ2_1
Aja_FoxQ2-1
Pmi_FoxQ2
Hpu_FoxQ2a
Spu_FoxQ2
Hpu_FoxQ2b
Sko_FoxQ2b
Cgi_FoxQ2-1
Las_FoxQ2B
Lan_FoxQ2-1
Pha_FoxQ2b
Dpo_FoxQ2-1
Dpo_FoxQ2-2
Ofu_FoxQ2-1
Ofu_FoxQ2-4
Ofu_FoxQ2-5
Ofu_FoxQ2-10
Ofu_FoxQ2-9
Ofu_FoxQ2-6
Ofu_FoxQ2-11
Sca_FoxQ2_2
Ofu_FoxQ2-7
Xbo_FoxQ2
Pca_FoxQ2
Dpo_FoxQ2-5
Dpo_FoxQ2-6
Nve_FoxQ2-1
Sko_FoxQ1
Bflo_FoxQ1
Ofu_FoxQ1
Spu_FoxQ1
0.3
B
FoxQ2III type
FoxQ2II-type
FoxQ2I-type
0.06
C
FoxQ2II-type
FoxQ2I-type
FoxQ2III type
0.06

Supplementary Fig. 3. Additional phylogenetic trees of FoxQ2 genes across metazoans.
A. Tree obtained with Maximum Likelihood analysis of the Forkhead domain in 47 species from 21 animal phyla. **B-C.** Neighbor joining tree topologies using full *FoxQ2* sequences (**B**) or only the Forkhead domain (**C**). In all trees, sequences are colored based on taxonomy: deuterostomes (red), spiralian (blue) and ecdysozoan (green) protostomes, xenacoelomorphs (yellow), cnidarians (purple), ctenoforans (cyan) or poriferans (orange). Gray-shaded boxes demarcate the three *FoxQ2* types, *FoxQ2I*, *FoxQ2II* and *FoxQ2III*. *FoxQ1* and *FoxP* sequences are used as outgroup. Full species names are listed in Supplementary Data 1. Scale bars indicate the number of amino acids substitutions per site.

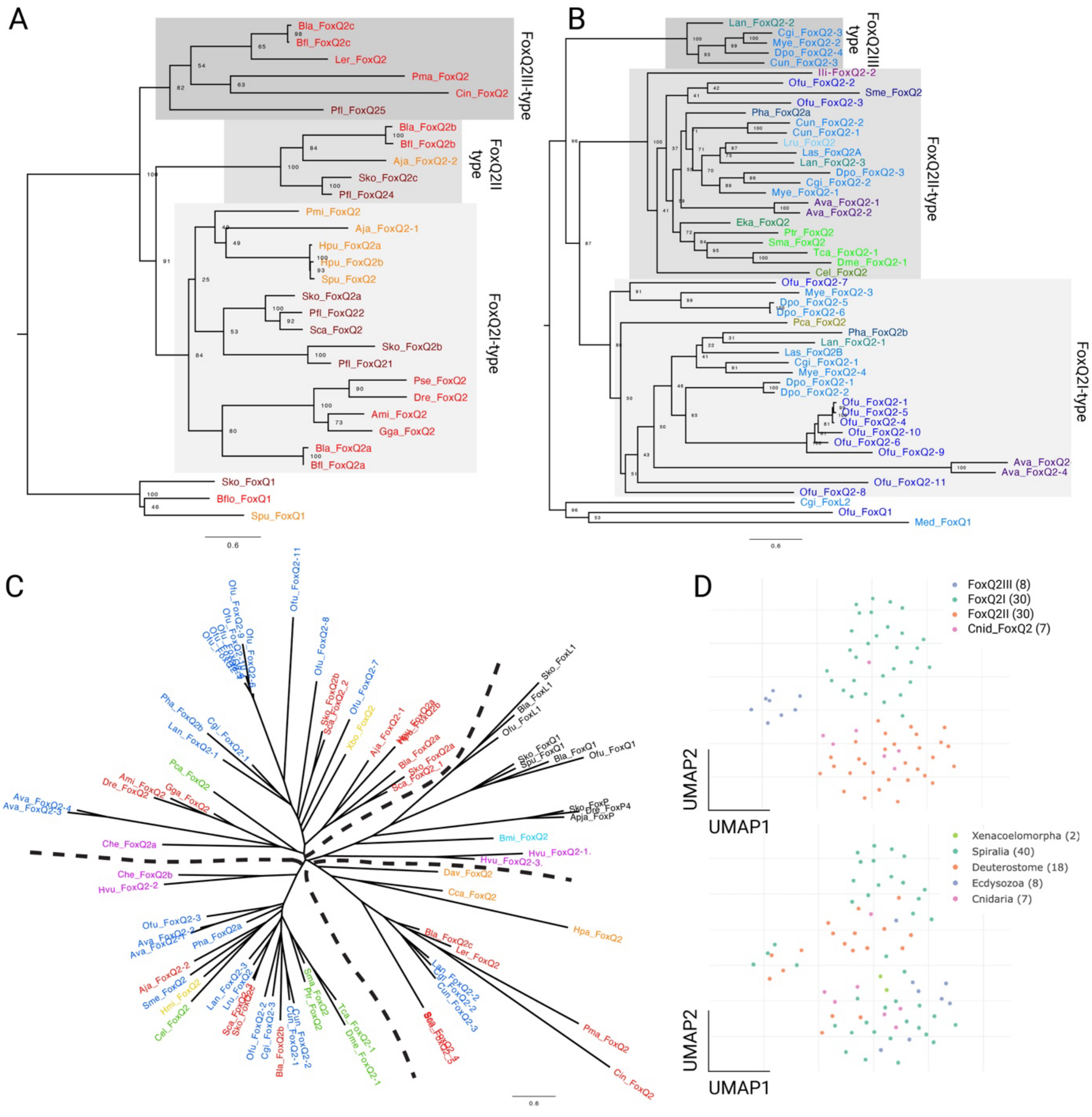

Supplementary Fig. 4. Additional phylogenetic comparisons of FoxQ2 genes.
A-B. Maximum Likelihood analysis considering only deuterostome (**A**) and protostome (**B**) species recover the same tree topology than the full metazoan phylogenetic tree. Sequences from different phyla are demarcated by different shades of red (A, deuterostomes), blue (B, spiralians) and green (B, ecdysozoa). Gray-shaded boxes demarcate the three *FoxQ2* types, *FoxQ2I*, *FoxQ2II* and *FoxQ2III*. **C.** Unrooted tree resulting from Maximum Likelihood analysis of full *FoxQ2* sequences, showing preservation of *FoxQ2I*, *FoxQ2II* and *FoxQ2III* types separated by dashed lines, and the difficult placement of cnidarian sequences due to high

divergence. Sequences are colored based on taxonomy: deuterostomes (red), spiralian (blue) and ecdysozoan (green) protostomes, xenacoelomorphs (yellow), cnidarians (purple). **D.** Result of uniform manifold approximation and projection (UMAP) dimensionality reduction using aligned forkhead domain of metazoan *FoxQ2* genes, colored by *FoxQ2* type (*FoxQ2I*, *FoxQ2II*, *FoxQ2III* and cnidarian *FoxQ2*) or by taxon, showing the presence of three separated clusters of sequences in accordance with phylogenetic analysis. Full species names are listed in Supplementary Data 1. Scale bars in A, B and C indicate the number of amino acids substitutions per site.

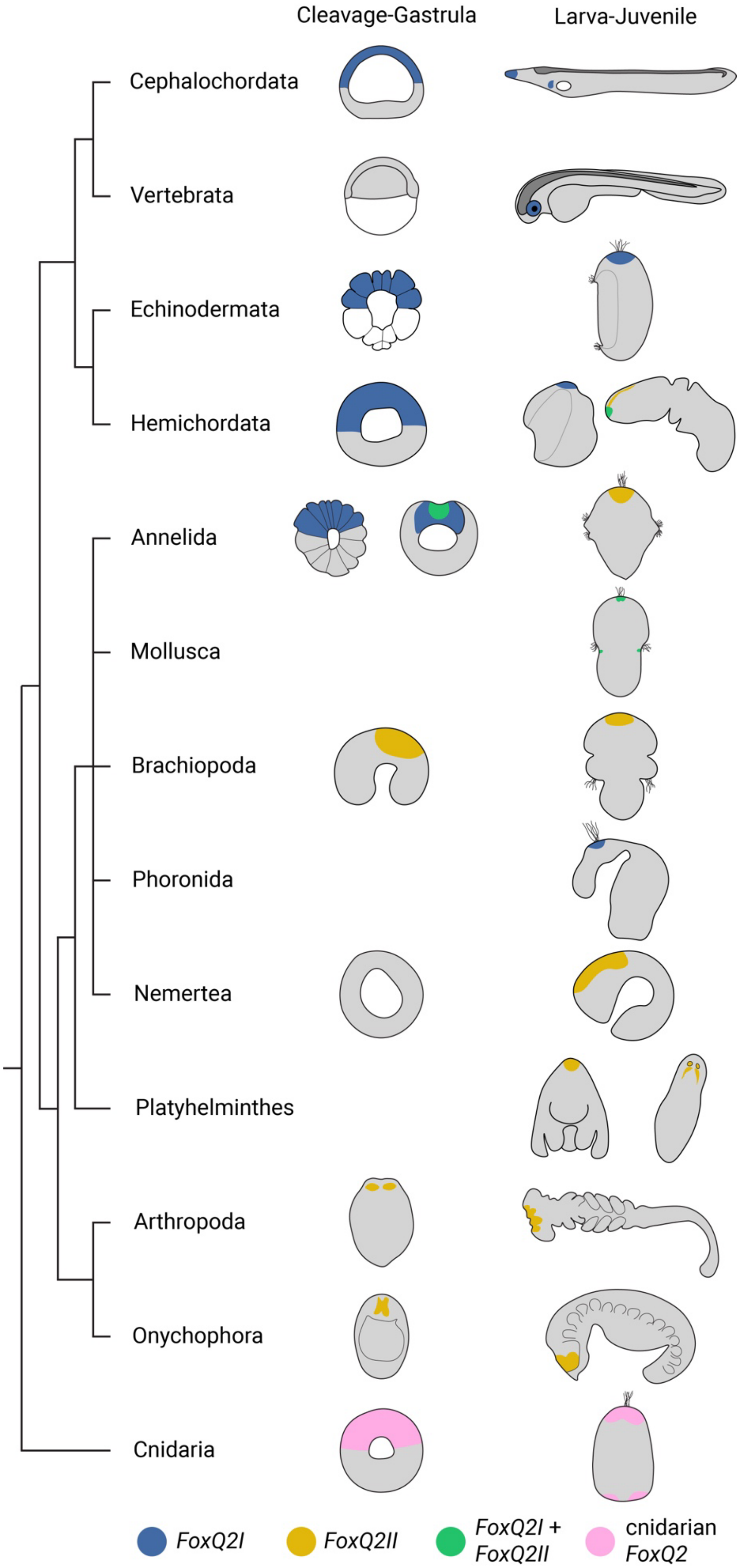

Cleavage-Gastrula
Larva-Juvenile
Cephalochordata
Vertebrata
Echinodermata
Hemichordata
Annelida
Mollusca
Brachiopoda
Phoronida
Nemertea
Platyhelminthes
Arthropoda
Onychophora
Cnidaria
FoxQ2I
FoxQ2II
FoxQ2I + FoxQ2II
cnidarian FoxQ2

Supplementary Fig. 5. Summary of *FoxQ2I* and *FoxQ2II* expression during animal development.

Schematic drawings of early and late developmental stages of representative species in which the expression of *FoxQ2I*-type (blue) and *FoxQ2II*-type (yellow) genes has been investigated. Due to the difficult positioning of *FoxQ2I*- and *FoxQ2II*-type genes in cnidarians, the developmental expression domain of *FoxQ2* genes in cnidarians has been colored separately.

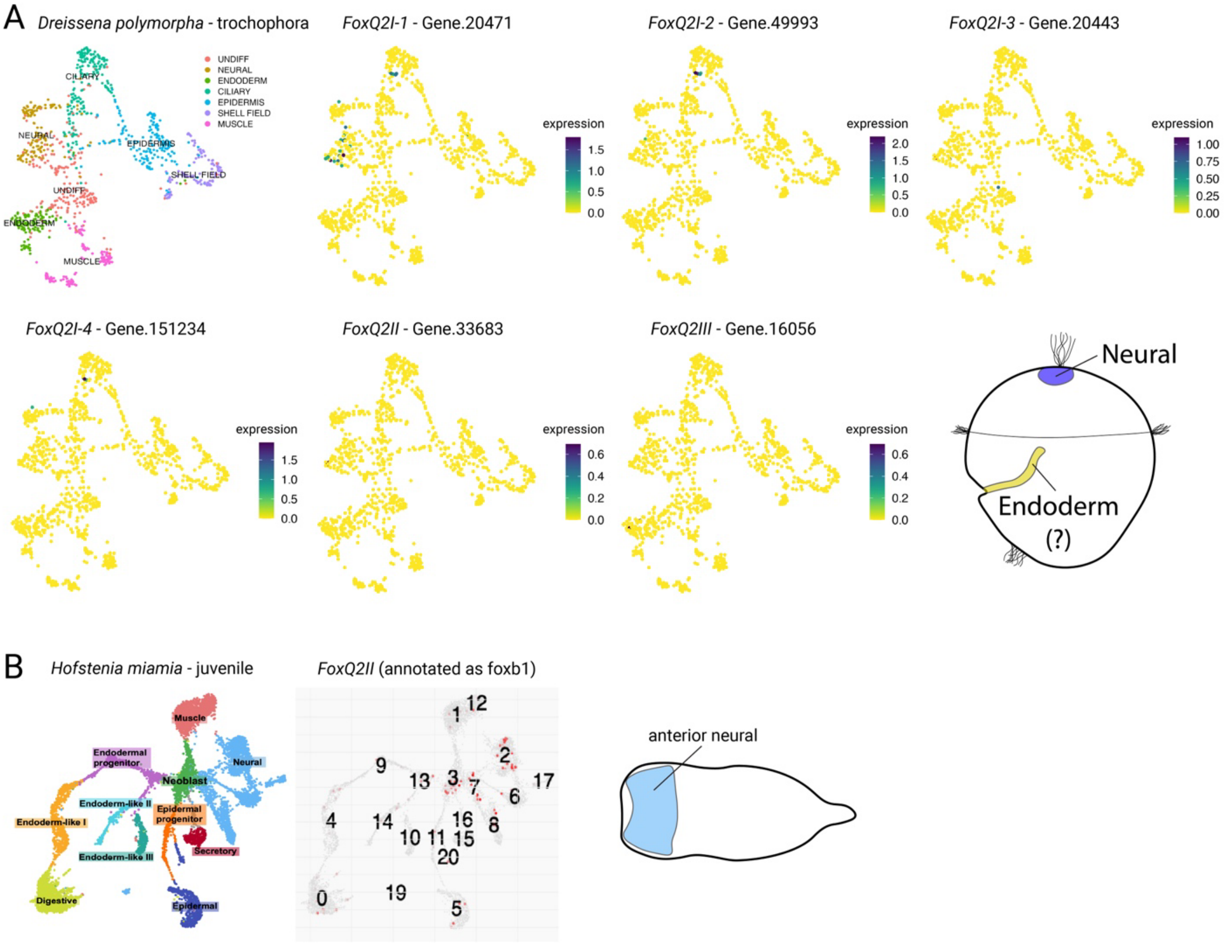

Supplementary Fig. 6. Analysis of *FoxQ2* expression in publicly available invertebrate single-cell RNAseq datasets.

A. Cluster annotation and expression of *FoxQ2I*, *FoxQ2II*, and *FoxQ2III* orthologs in the early trochophore larva of the bivalve mollusk *Dreissena polymorpha* (data from [1]), showing expression of one *FoxQ2I*-type gene in neurons, sparse expression of two *FoxQ2I* and one *FoxQ2II* gene in ciliated cells, and rare expression of *FoxQ2III* in the endoderm. A schematic representation of the trochophora larva highlights the localization of neural and endodermal cells. UMAP expression plots show normalized UMI counts for each gene. **B.** Expression of *FoxQ2II*-type gene in the juvenile stage of the acoel *Hofstenia miamia* (data from [2]), showing positive cells in the neural and neoblast clusters. The *FoxQ2II*-positive neural clusters 2 and 7 have been mapped by the original authors as anterior neural clusters, as represented in the schematic drawing of *H. miamia* juvenile. UMAP expression plots show normalized UMI counts for each gene.

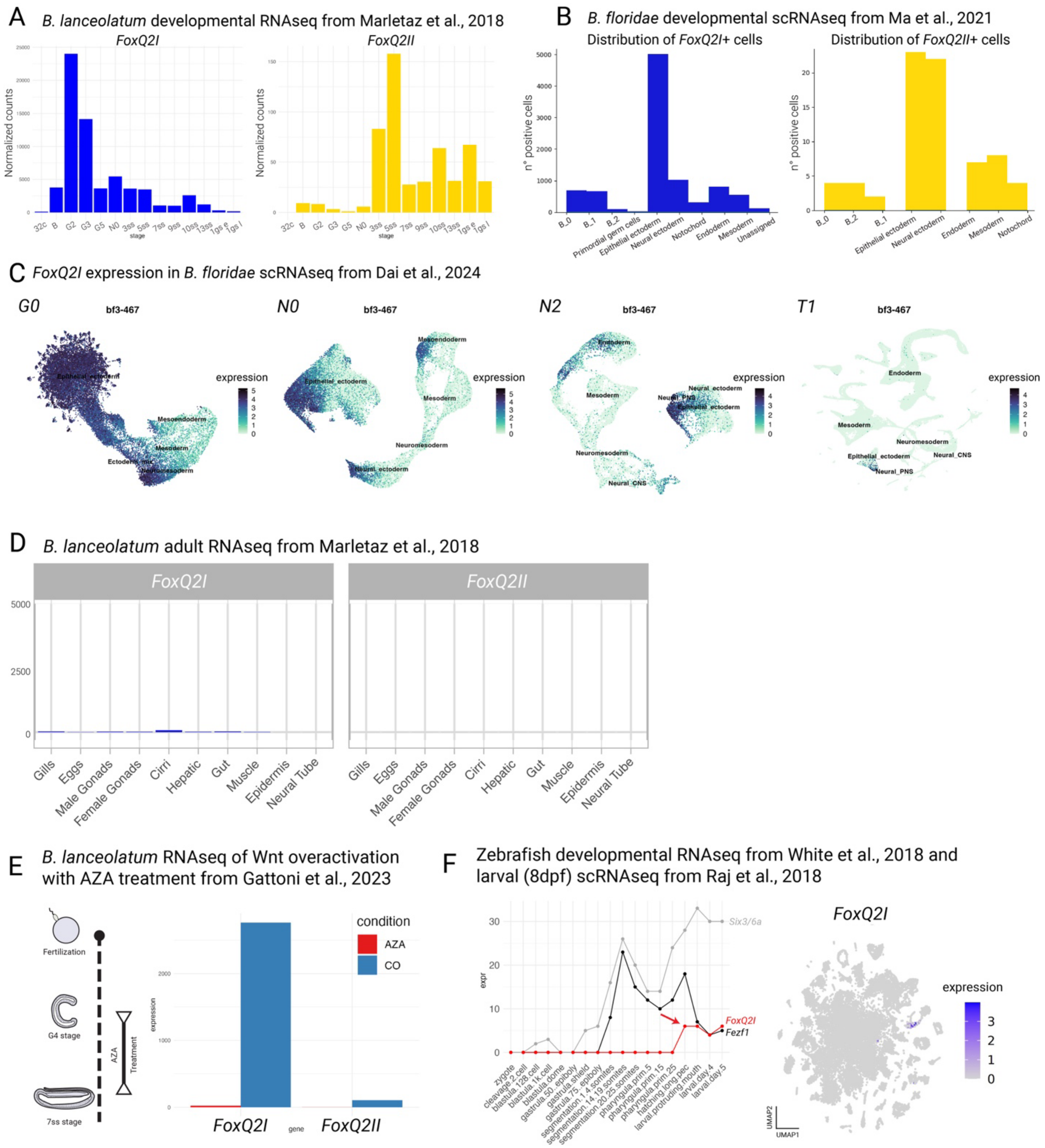

Supplementary Fig. 7. Additional transcriptomic analyses of amphioxus and zebrafish *FoxQ2I*- and *FoxQ2II-types* in support of *in situ* hybridization data.

A. Developmental expression levels of *FoxQ2I* (blue), *FoxQ2II* (yellow) in *Branchiostoma lanceolatum* from bulk RNAseq[3], indicating activation of *FoxQ2I* at the blastula stage and *FoxQ2II* at the early neurula stage. **B.** Distribution of *FoxQ2I* and *FoxQ2II* in single-cell RNA sequencing (scRNAseq) data from *B. floridae* development[4]. The distribution is calculated as

the number of positive cells (y axis) in each annotated cluster (x axis), and shows expression of *FoxQ2I* and FoxQ2II in neural and non-neural ectoderm. **C.** This result is further confirmed by the distribution of *FoxQ2I*-positive cells in a scRNAseq dataset from *B. floridae* across development[5], from early gastrula to early larva. *FoxQ2I* is initially expressed in epithelial ectoderm and anterior neural endoderm, but expression remains only in the epithelial ectoderm at larval stage. **D.** Expression level (y axis) of *FoxQ2I* and *FoxQ2II* in adult tissues of *B. lanceolatum* from bulk RNAseq[3], showing that expression of both genes is highly reduced in adults. **E.** Significant differential expression of *FoxQ2I* and *FoxQ2II* in control (blue) and Azakenpaullone treated (red) embryos (data from [6]), demonstrating loss of expression for both genes following pharmacological overactivation of Wnt signalling. **F.** Bulk RNAseq dataset of zebrafish development[7] shows that *FoxQ2I* starts to be expressed during the beginning of larval stages, much later than the peak of expression of anterior neuroectoderm genes such as *Six3a* and *Fezf1*. scRNAseq data of 8 days old zebrafish larvae[8] shows expression of *FoxQ2* restricted to blue cones and photoreceptor precursors. All UMAP expression plots show normalized UMI counts for each gene.

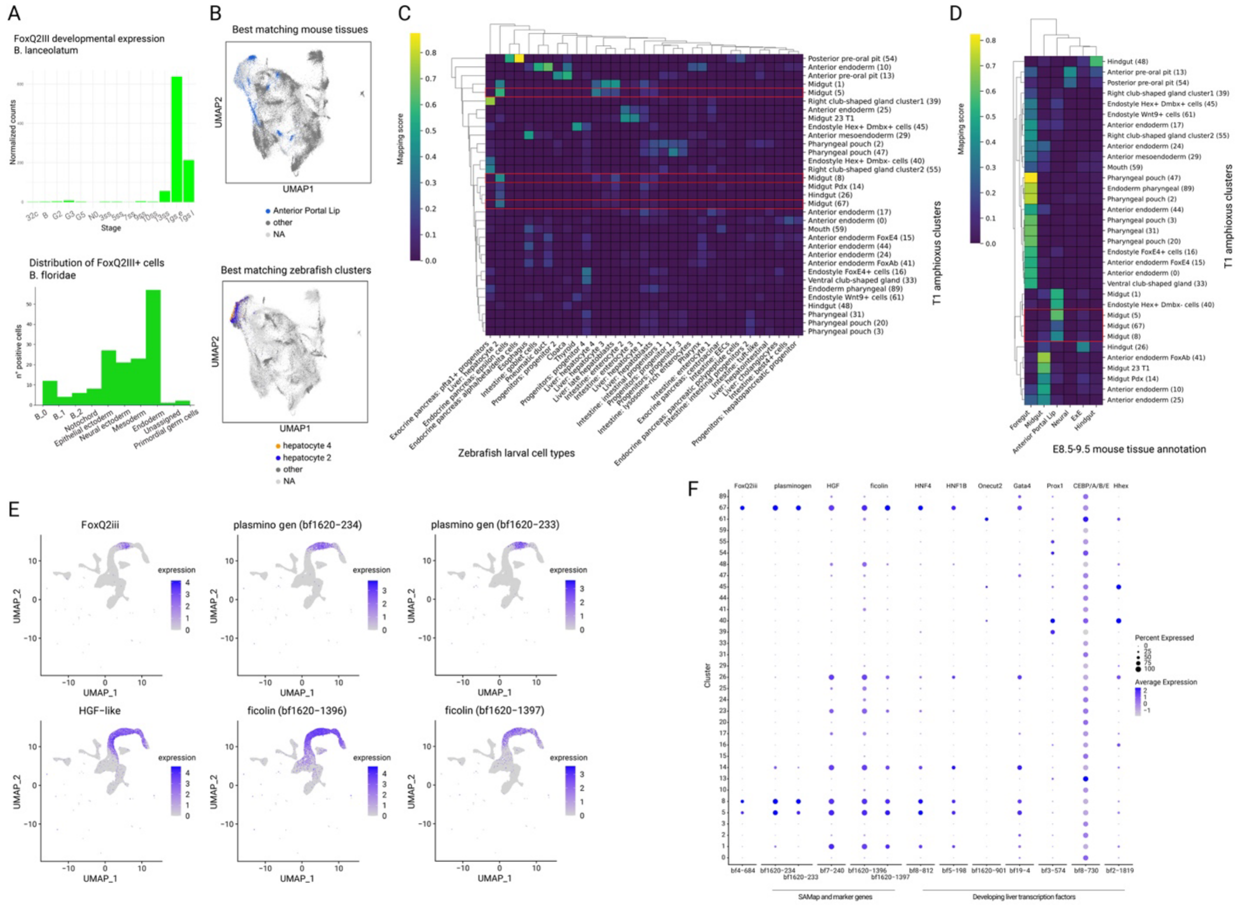

Supplementary Fig. 8. Cross-species comparative analysis of amphioxus larval *FoxQ2III*-positive gut cells shows molecular similarity with developing vertebrate liver cells.
A. Developmental expression levels of *FoxQ2III* in *Branchiostoma lanceolatum* from bulk RNAseq[3], and number of *FoxQ2III*-positive cells per tissue in *B. floridae* developmental single-cell RNA sequencing (scRNAseq) data, showing expression in the endoderm from early larva stage. **B**. UMAP embeddings of embryonic SAMap multispecies integration of amphioxus[5], mouse[9] and zebrafish[10] embryos, with cells colored by best matching tissues from mouse and cell types from zebrafish. **C.** Heatmap of all amphioxus-mouse pairwise mapping scores (fraction of cross-species mutual nearest neighbors). The *FoxQ2III*-expressing midgut clusters 1, 5, and 67 and indicated in bold. **D**. Heatmap of all amphioxus-zebrafish pairwise mapping scores. **E.** UMAP embeddings of endoderm from T1 amphioxus scRNA-seq, as published in [5]. Gene expression shown as normalized UMI counts is shown for *FoxQ2III*, the top marker gene *FoxQ2III* clusters (bf1260-234), and top genes driving cross-species similarity nominated by SAMap. **F.** Dot plot of these genes, in addition to transcription factors that control liver development. Circles are scaled to the fraction of expressing cells per cluster and colored by mean expression.

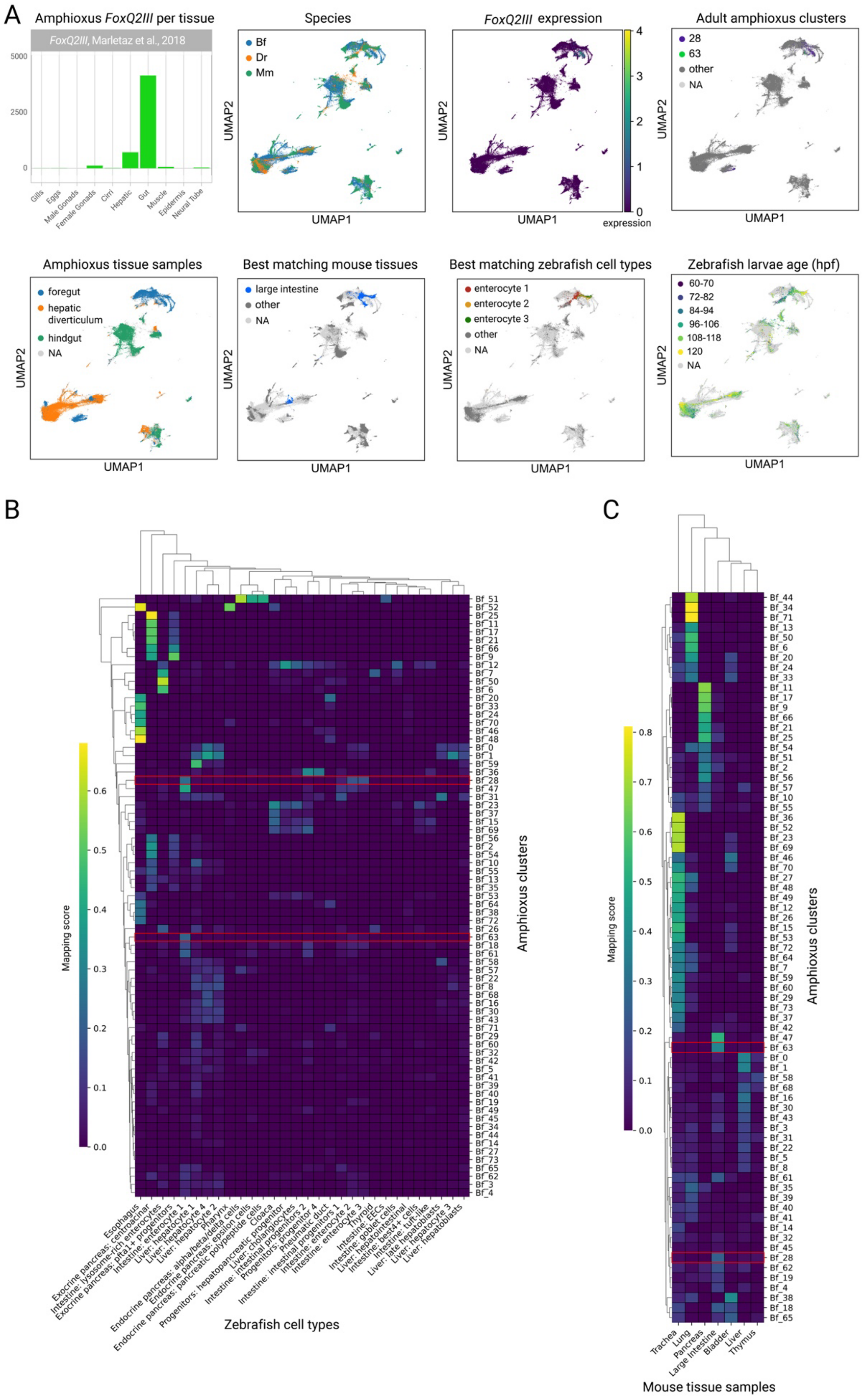

A
Amphioxus FoxQ2III per tissue
FoxQ2III, Marletaz et al., 2018
5000
2500
0
Gills
Eggs
Male Gonads
Female Gonads
Cirri
Hepatic
Gut
Muscle
Epidermis
Neural Tube
Species
Bf
Dr
Mm
FoxQ2III expression
expression
Adult amphioxus clusters
28
63
other
NA
Amphioxus tissue samples
foregut
hepatic diverticulum
hindgut
NA
Best matching mouse tissues
large intestine
other
NA
Best matching zebrafish cell types
enterocyte 1
enterocyte 2
enterocyte 3
other
NA
Zebrafish larvae age (hpf)
60-70
72-82
84-94
96-106
108-118
120
NA
UMAP1
UMAP2
B
Mapping score
Amphioxus clusters
Esophagus
Exocrine pancreas: centroacinar
Intestine: lysosome-rich enterocytes
Exocrine pancreas: ptf1a+ progenitors
Intestine: enterocyte 1
Liver: hepatocyte 1
Liver: hepatocyte 4
Liver: hepatocyte 2
Pharynx
Endocrine pancreas: alpha/beta/delta cells
Endocrine pancreas: epsilon cells
Endocrine pancreas: pancreatic polypeptide cells
Cloaca
Progenitors: hepatopancreatic progenitor
Liver: cholangiocytes 2
Intestine: intestinal progenitors 2
Progenitors: progenitor 4
Pneumatic duct
Intestine: intestinal progenitors 1
Intestine: enterocyte 2
Intestine: enterocyte 3
Thyroid
Intestine: EECs
Intestine: goblet cells
Liver: hepatointestinal
Intestine: best4+ cells
Intestine: tuft-like
Liver: late hepatoblasts
Liver: hepatocyte 3
Liver: hepatoblasts
Zebrafish cell types
C
Mapping score
Amphioxus clusters
Trachea
Lung
Pancreas
Large Intestine
Bladder
Liver
Thymus
Mouse tissue samples

Supplementary Fig. 9. *FoxQ2III* expression in adult amphioxus and cross-species integrations of adult amphioxus gut tube single-cell RNA sequencing with vertebrate datasets.

A. Bulk RNA-seq of *Branchiostoma lanceolatum* adults[3] showing maintenance of high *FoxQ2III* expression in the adult digestive system, and UMAP embeddings of SAMap multispecies integration of amphioxus adult gut[11], mouse adult gut tissues[12] and zebrafish[10] colored by selected metadata: species, *FoxQ2III* expression (normalized UMI counts), amphioxus *FoxQ2III*-expressing clusters, best matching zebrafish cell types, best matching mouse tissues, amphioxus (*Branchiostoma floridae*) sample of origin, and zebrafish larval age. **B.** Heatmap of all amphioxus-zebrafish pairwise mapping scores. The *FoxQ2III*-expressing midgut clusters 28 and 67 are highlighted in red boxes. **C**. Heatmap of all amphioxus-mouse pairwise mapping scores. The FoxQ2iii-expressing midgut clusters 28 and 67 are highlighted in red boxes.

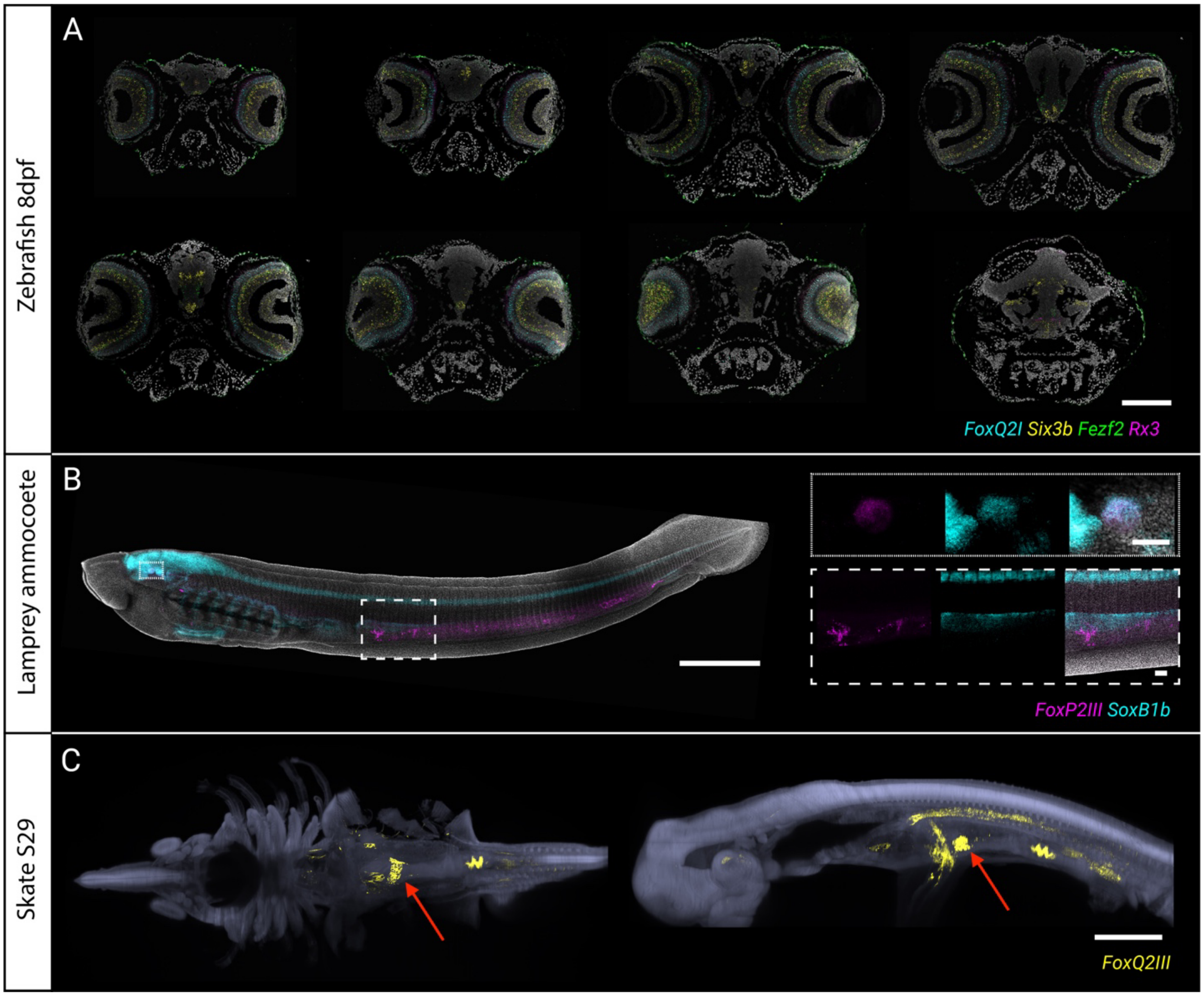

Supplementary Fig. 10. Additional panels showing expression pattern of vertebrate *FoxQ2* orthologs.

A. Co-detection of *FoxQ2I* with conserved anterior neuroectoderm markers *Six3b*, *Fezf2* and *Rx3* in 8dpf zebrafish larvae. *FoxQ2I* is not expressed in the brain, where all other genes collectively label distinct forebrain cell types, but is expressed in the retina photoreceptor layer. **B.** Whole-mount *in situ* HCR of lamprey ammocoete larva showing expression of *FoxQ2III* and *SoxB1b* in the gut (dashed box) and the eye (dotted box). **C.** Whole-mount S29 skate embryo labelled with *FoxQ2III* before the nuclear masking used in fig. 4, showing aspecific expression in the body cavity. The real expression in the midgut is marked by a red arrow. Scalebars are 100μm for A and insets in B, 1 mm for B and C.

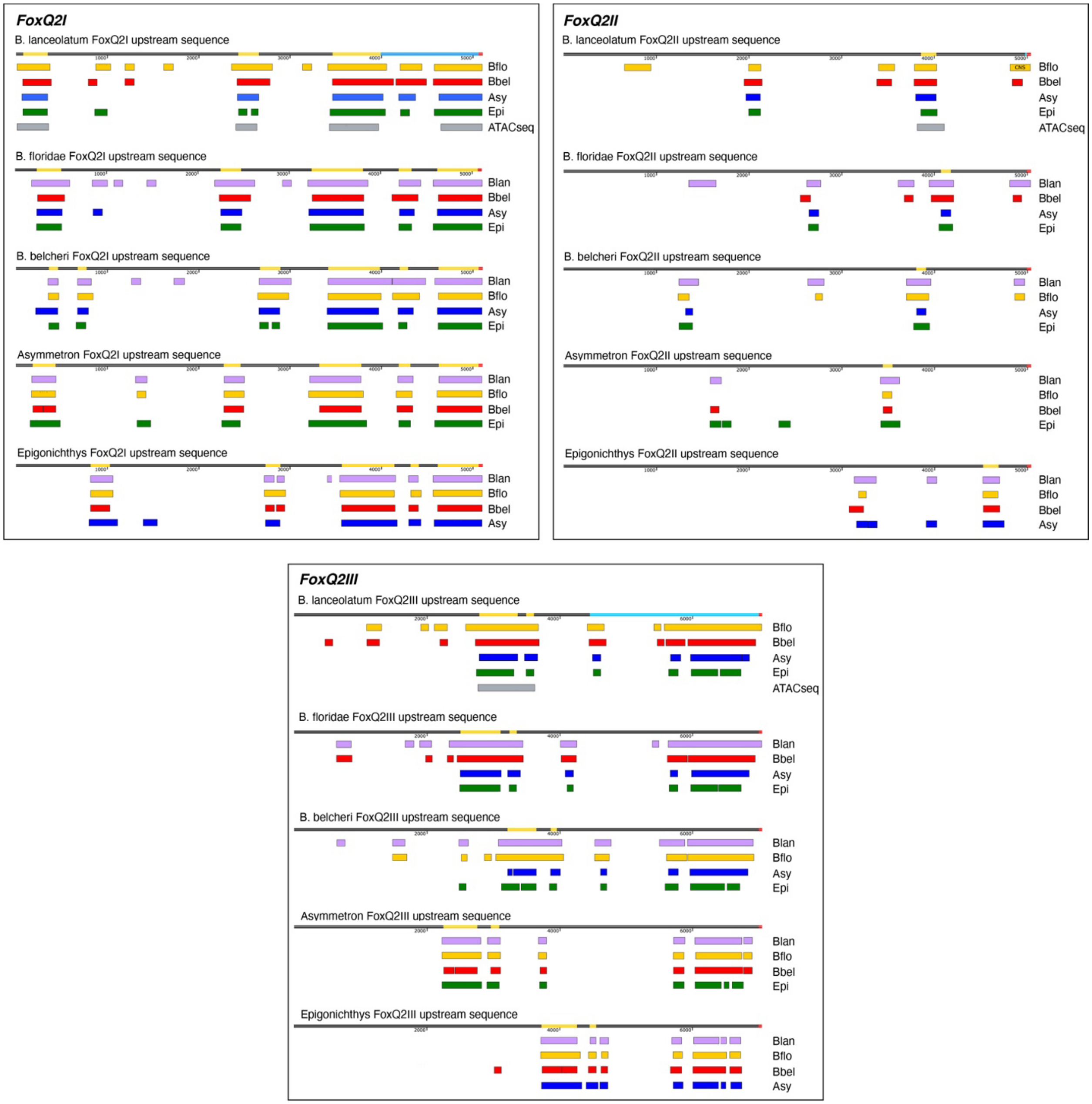

Supplementary Fig. 11. Conserved Non-Coding Sequences for FoxQ2I, FoxQ2II and FoxQ2III in cephalochordates.

Conserved nucleotide sequences upstream of the three *FoxQ2* genes (*FoxQ2I*, *Fox2b*, *FoxQ2III*) in five cephalochordate species identified using mVISTA. Each *FoxQ2* upstream sequence for each species was compared to all other four species. For each sequence (represented as a black double line), conserved non-coding sequences (CNCSs) are highlighted in yellow, the

starting codon is in red and the 5′UTR region is in light blue. For each species, the conserved regions are indicated with violet (*Branchiostoma lanceolatum*), yellow (*B. floridae*), red (*B. belcheri*), blue (*Asymmetron*) and green (*Epigonichthys*) bars. CNCSs are defined as the regions that show conservation across all five species. For *B. lanceolatum*, the open chromatin regions identified by ATACseq are marked by a grey bar, showing that they correspond to the CNCS regions.

Supplementary References

1. Salamanca-Díaz, D. A., Schulreich, S. M., Cole, A. G. & Wanninger, A. Single-Cell RNA Sequencing Atlas From a Bivalve Larva Enhances Classical Cell Lineage Studies. *Front. Ecol. Evol.* **9**, 783984 (2022).
2. Hulett, R. E. *et al.* Acoel single-cell atlas reveals expression dynamics and heterogeneity of adult pluripotent stem cells. *Nat Commun* **14**, 2612 (2023).
3. Marlétaz, F. *et al.* Amphioxus functional genomics and the origins of vertebrate gene regulation. *Nature* **564**, 64–70 (2018).
4. Ma, P. *et al.* Joint profiling of gene expression and chromatin accessibility during amphioxus development at single-cell resolution. *Cell Reports* **39**, (2022).
5. Dai, Y. *et al.* Evolutionary origin of the chordate nervous system revealed by amphioxus developmental trajectories. *Nat Ecol Evol* (2024) doi:10.1038/s41559-024-02469-7.
6. Gattoni, G., Keitley, D., Sawle, A. & Benito-Gutiérrez, E. An ancient apical patterning system sets the position of the forebrain in chordates. *Sci. Adv.* **11**, eadq4731 (2025).
7. White, R. J. *et al.* A high-resolution mRNA expression time course of embryonic development in zebrafish. *eLife* **6**, e30860 (2017).
8. Raj, B. *et al.* Emergence of Neuronal Diversity during Vertebrate Brain Development. *Neuron* **108**, 1058-1074.e6 (2020).
9. Li, K.-R. *et al.* Spatiotemporal and genetic cell lineage tracing of endodermal organogenesis at single-cell resolution. *Cell* **188**, 796-813.e24 (2025).

10. Sur, A. *et al.* Single-cell analysis of shared signatures and transcriptional diversity during zebrafish development. *Developmental Cell* **58**, 3028-3047.e12 (2023).

11. Dai, Y. *et al.* Single-cell profiling of the amphioxus digestive tract reveals conservation of endocrine cells in chordates. *Sci. Adv.* **10**, (2024).

12. The Tabula Muris Consortium *et al.* Single-cell transcriptomics of 20 mouse organs creates a Tabula Muris. *Nature* **562**, 367–372 (2018).
