# Supplementary material for: Evolutionary dynamics of FoxQ2 transcription factors across metazoans reveals three ancient paralogs
Source: Commun Biol. 2025 Dec 21;9:98. doi: 10.1038/s42003-025-09368-y (PMC12828049; doi:10.1038/s42003-025-09368-y)
Supplement: Supplementary file 2 — Description of Additional Supplementary Materials [file 42003_2025_9368_MOESM2_ESM.pdf]

Description of Additional Supplementary Files

File name: Supplementary Data 1

Description: Complete list of FoxQ2 genes in 70 metazoan species, with division into a, b and c types and new nomenclature

File name: Supplementary Data 2

Description: Macro-synteny analysis results for 29 metazoan species and two non-metazoan Opisthokonta outgroups.

File name: Supplementary Data 3

Description: SAMap-generated top marker gene pairs for amphioxus-vertebrate clusters with a mapping score >0.2 for embryonic and adult single-cell RNA sequencing integrations

File name: Supplementary Data 4

Description: Analysis of Conserved Non-Coding Sequences (CNCSs) in cephalochordates. The table contains:

- Sequences of each CNCS in B. lanceolatum

- List of conserved amphioxus transcription

factors that have identified transcription

factor binding sites (TFBSs) in each CNCS

and that are expressed together with each

FoxQ2 gene

- List of transcription factor families

identified in the study
