# Supplementary material for: Evolutionary dynamics of FoxQ2 transcription factors across metazoans reveals three ancient paralogs
Source: Commun Biol. 2025 Dec 21;9:98. doi: 10.1038/s42003-025-09368-y (PMC12828049; doi:10.1038/s42003-025-09368-y)
Supplement: Supplementary file 4 — Reporting Summary [file 42003_2025_9368_MOESM4_ESM.pdf]

nature portfolio

Corresponding author(s): Elia Benito-Gutiérrez
Giacomo Gattoni

Last updated by author(s): 20.10.2025

Reporting Summary

Nature Portfolio wishes to improve the reproducibility of the work that we publish. This form provides structure for consistency and transparency in reporting. For further information on Nature Portfolio policies, see our Editorial Policies and the Editorial Policy Checklist.

Statistics

For all statistical analyses, confirm that the following items are present in the figure legend, table legend, main text, or Methods section.

n/a	Confirmed	
☒	☐	The exact sample size (*n*) for each experimental group/condition, given as a discrete number and unit of measurement
☒	☐	A statement on whether measurements were taken from distinct samples or whether the same sample was measured repeatedly
☒	☐	The statistical test(s) used AND whether they are one- or two-sided<br>*Only common tests should be described solely by name; describe more complex techniques in the Methods section.*
☒	☐	A description of all covariates tested
☒	☐	A description of any assumptions or corrections, such as tests of normality and adjustment for multiple comparisons
☒	☐	A full description of the statistical parameters including central tendency (e.g. means) or other basic estimates (e.g. regression coefficient) AND variation (e.g. standard deviation) or associated estimates of uncertainty (e.g. confidence intervals)
☒	☐	For null hypothesis testing, the test statistic (e.g. *F*, *t*, *r*) with confidence intervals, effect sizes, degrees of freedom and *P* value noted<br>*Give P values as exact values whenever suitable.*
☒	☐	For Bayesian analysis, information on the choice of priors and Markov chain Monte Carlo settings
☒	☐	For hierarchical and complex designs, identification of the appropriate level for tests and full reporting of outcomes
☒	☐	Estimates of effect sizes (e.g. Cohen's *d*, Pearson's *r*), indicating how they were calculated

Our web collection on statistics for biologists contains articles on many of the points above.

Software and code

Policy information about availability of computer code

Data collection	n/a
Data analysis	All code used in this project can be found in the project Github repository, available at https://github.com/eBGLab/FoxQ2_Evolution.

For manuscripts utilizing custom algorithms or software that are central to the research but not yet described in published literature, software must be made available to editors and reviewers. We strongly encourage code deposition in a community repository (e.g. GitHub). See the Nature Portfolio guidelines for submitting code & software for further information.

Data

Policy information about availability of data

All manuscripts must include a data availability statement. This statement should provide the following information, where applicable:

- Accession codes, unique identifiers, or web links for publicly available datasets
- A description of any restrictions on data availability
- For clinical datasets or third party data, please ensure that the statement adheres to our policy

All data required to evaluate the conclusions in the paper are present in the paper and/or the Supplementary Materials. The code used to generate figures and data is available at https://github.com/eBGLab/FoxQ2_Evolution and on Zenodo

Research involving human participants, their data, or biological material

Policy information about studies with human participants or human data. See also policy information about sex, gender (identity/presentation), and sexual orientation and race, ethnicity and racism.

Reporting on sex and gender	n/a
Reporting on race, ethnicity, or other socially relevant groupings	n/a
Population characteristics	n/a
Recruitment	n/a
Ethics oversight	n/a

Note that full information on the approval of the study protocol must also be provided in the manuscript.

Field-specific reporting

Please select the one below that is the best fit for your research. If you are not sure, read the appropriate sections before making your selection.

☒ Life sciences ☐ Behavioural & social sciences ☐ Ecological, evolutionary & environmental sciences

For a reference copy of the document with all sections, see nature.com/documents/nr-reporting-summary-flat.pdf

Life sciences study design

All studies must disclose on these points even when the disclosure is negative.

Sample size	Overall, this study used ~50 amphioxus embryos,
Data exclusions	No data or sample was excluded from the analysis
Replication	For in situ hybridization chain reaction, experiments were repeated 2 times for each marker examined. For each round of in situ hybridization chain reaction, ~5 samples per stage per experiment were examined to ensure consistency of the staining, and at least 2-3 samples per stage per experiment and condition were imaged at high resolution with confocal microscopy.
Randomization	For in situ hybridization chain reaction, embryos and larvae were randomly selected for all the experiments.
Blinding	Blinding was not applicable for in situ hybridization chain reaction and the analysis

Reporting for specific materials, systems and methods

We require information from authors about some types of materials, experimental systems and methods used in many studies. Here, indicate whether each material, system or method listed is relevant to your study. If you are not sure if a list item applies to your research, read the appropriate section before selecting a response.

Materials & experimental systems

n/a	Involved in the study	
☒	☐	Antibodies
☒	☐	Eukaryotic cell lines
☒	☐	Palaeontology and archaeology
☐	☒	Animals and other organisms
☒	☐	Clinical data
☒	☐	Dual use research of concern
☒	☐	Plants

Methods

n/a	Involved in the study	
☒	☐	ChIP-seq
☒	☐	Flow cytometry
☒	☐	MRI-based neuroimaging

Animals and other research organisms

Policy information about studies involving animals; ARRIVE guidelines recommended for reporting animal research, and Sex and Gender in Research

Laboratory animals	The study did not involve laboratory animals. The study used embryos and larvae of amphioxus, lamprey, skate, zebrafish and chick as detailed below.
Wild animals	Adult European amphioxus (Branchiostoma lanceolatum) were collected from the sand in Banyuls-sur- Mer, France, and transported to Cambridge, UK, where they were kept in a custom-made facility at the Department of Zoology. Adult individuals were spawned by heat-shock and then reintroduced to the animal facility. Embryos were generated by in vitro fertilization, grown in artificial sea water and fixed at the desired stages.<br><br>Adult sea lamprey (Petromyzon marinus) were collected from the Hammond Bay Biological Station, Millersburg, MI, and shipped to Northwestern University. Embryos and larvae were obtained by in vitro fertilization and fixed at the desired stages.<br><br>Little skate (Leucoraja erinacea) embryos were obtained from the Marine Resources Center at the Marine Biological Laboratory (MBL) in Woods Hole, MA, U.S.A., and reared to stage 29 before fixation.
Reporting on sex	The sex of adult animals can be identified by looking at the gonads, while the sex of embryos and larvae cannot be determined, and it does not influence the experiments carried out in this work.
Field-collected samples	Adult amphioxus were kept in a custom-made facility with close circulation of artificial sea water at a temperature that varied between 9-13°C through the year. The summer photoperiod was 14h day-10h night, while the winter photoperiod was 10h day-14h night.
Ethics oversight	Experiments using larval and adult zebrafish were conducted according to protocols approved by the Institutional Animal Care and Use Committees in facilities accredited by the Association for Assessment and Accreditation of Laboratory Animal Care International (AAALAC). The use of tissue samples for this study was reviewed and ethically approved by the University of Cambridge Animal Welfare and Ethical Review Body (AWERB) Committee.<br><br>All procedures on lampreys were approved by Northwestern University's Institutional Animal Care and Use Committee (IACUC A3283-01).<br><br>All skate experiments were conducted according to protocols approved by the Institutional Animal Care and Use Committee of the Marine Biological Laboratory (Woods Hole, MA, USA).

Note that full information on the approval of the study protocol must also be provided in the manuscript.

Plants

Seed stocks	n/a
Novel plant genotypes	n/a
Authentication	n/a
